# Processing of the Terminal Alpha-1,2-Linked Mannose Residues From Oligomannosidic *N*-Glycans Is Critical for Proper Root Growth

**DOI:** 10.3389/fpls.2018.01807

**Published:** 2018-12-06

**Authors:** Christiane Veit, Julia König, Friedrich Altmann, Richard Strasser

**Affiliations:** ^1^Department of Applied Genetics and Cell Biology, University of Natural Resources and Life Sciences, Vienna, Austria; ^2^Department of Chemistry, University of Natural Resources and Life Sciences, Vienna, Austria

**Keywords:** endoplasmic reticulum, Golgi apparatus, protein glycosylation, *N*-glycosylation, glycoprotein, mannosidase

## Abstract

*N*-glycosylation is an essential protein modification that plays roles in many diverse biological processes including protein folding, quality control and protein interactions. Despite recent advances in characterization of the *N*-glycosylation and *N*-glycan processing machinery our understanding of *N*-glycosylation related processes in plant development is limited. In *Arabidopsis thaliana*, failure of mannose trimming from oligomannosidic *N*-glycans in the endoplasmic reticulum (ER) and *cis*/medial-Golgi leads to a defect in root development in the *mns123* triple mutant. Here, we show that the severe root phenotype of *mns123* is restored in asparagine-linked glycosylation (ALG)-deficient plants with distinct defects in the biosynthesis of the lipid-linked oligosaccharide precursor. The root growth of these ALG-deficient plants is not affected by the α-mannosidase inhibitor kifunensine. Genetic evidence shows that the defect is uncoupled from the glycan-dependent ER-associated degradation (ERAD) pathway that removes misfolded glycoproteins with oligomannosidic *N*-glycans from the ER. Restoration of mannose trimming using a *trans*-Golgi targeted α-mannosidase suppresses the defect of *mns123* roots. These data suggest that processing of terminal mannose residues from oligomannosidic *N*-glycans is important for an unknown late-Golgi or post-Golgi process that is implicated in proper root formation.

## Introduction

**FIGURE 1:**
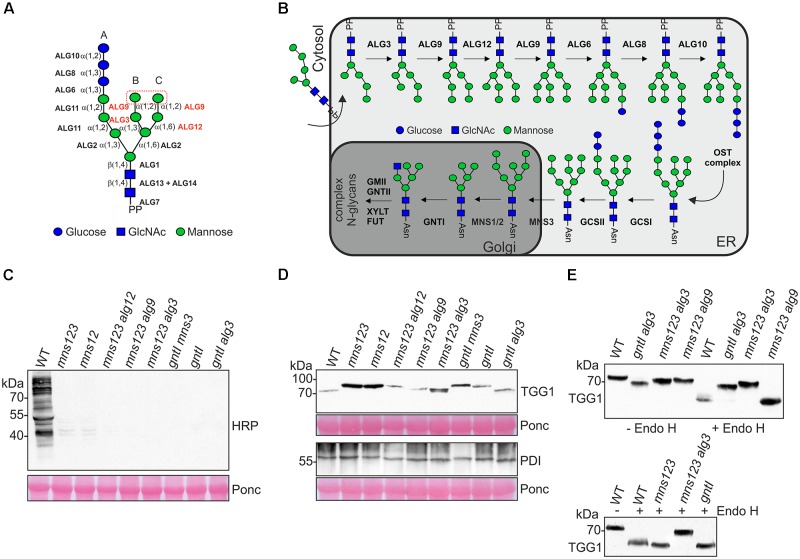
**(A)** Schematic illustration of the preassembled dolichol pyrophosphate-linked oligosaccharide precursor. The ALG glycosyltransferases required for the precursor biosynthesis are shown. The ALGs involved in the transfer of mannose residues in the lumen of the ER and the two terminal mannose residues on the B- and C-branches are highlighted in red. The symbol representation for glycans is drawn according to the guidelines from the Consortium for Functional Glycomics. **(B)** Illustration of *N*-glycan biosynthesis and processing pathways in wild-type. For the biosynthesis pathway only the steps in the lumen of the ER are shown. The following abbreviations are used for the enzymes: ALG3, α1,3-mannosyltransferase; ALG6, α1,3-glucosyltransferase; ALG8, α1,3-glucosyltransferase; ALG9, α1,2-mannosyltransferase; ALG10, α1,2-glucosyltransferase; ALG12, α1,6-mannosyltransferase; OST, oligosaccharyltransferase; GCSI, α-glucosidase I; GCSII, α-glucosidase II; MNS3, ER-α-mannosidase I; MNS1/MNS2, Golgi-α-mannosidase I; GNTI, *N*-acetylglucosaminyltransferase I; GMII, Golgi α-mannosidase II; GNTII, *N*-acetylglucosaminyltransferase II; XYLT, β1,2-xylosyltransferase; FUT, core α1,3-fucosyltransferase. Asn, asparagine of the *N*-glycosylation site (consensus: Asn-X-Ser/Thr). PP, dolichol pyrophosphate. **(C)** Immunoblot analysis with antibodies against complex *N*-glycans. A positive signal is only observed in wild-type Col-0 (WT) carrying processed complex *N*-glycans with β1,2-xylose and core α1,3-fucose residues. Ponceau S (Ponc) staining of the membrane is shown as a loading control. **(D)** Immunoblot analysis with antibodies directed against the glycoproteins TGG1 or PDI. **(E)** Endo H digestion of protein extracts and immunoblot analysis.

*N*-glycosylation of proteins is an essential co- and posttranslational modification in eukaryotes. During *N*-glycosylation a preassembled lipid-linked oligosaccharide is transferred *en bloc* to an asparagine residue that is present in the consensus sequence motif Asn-X-Ser/Thr of a polypeptide (Aebi, 2013). Assembly of the lipid-linked oligosaccharide occurs in an ordered stepwise manner by ALG (asparagine linked glycosylation) enzymes. The first steps of the lipid-linked oligosaccharide biosynthesis take place on the cytosolic side of the endoplasmic reticulum (ER) membrane. The synthesized Man_5_GlcNAc_2_-dolichol pyrophosphate is transported across the ER membrane and used by a series of different ALGs as an acceptor substrate. In the ER lumen, the first mannose residue is transferred by the α1,3-mannosyltransferase ALG3. Three additional mannose residues are attached to the B- and C-branches of the lipid-linked precursor by the α1,6-mannosyltransferase ALG12 and the α1,2-mannosyltransferase ALG9 (Figures 1A,B). The biosynthesis of the dolichol-linked oligosaccharide is completed by the successive addition of three glucose residues catalyzed by the glucosyltransferases ALG6, ALG8, and ALG10. The oligosaccharyltransferase complex transfers the fully assembled oligosaccharide to asparagine residues of newly synthesized proteins (Strasser, 2016).

*N*-glycan processing in the ER starts by removal of terminal glucose residues from the transferred oligosaccharide. The oligomannosidic *N*-glycans are trimmed further by specific α-mannosidases (MNS1, MNS2, and MNS3). MNS3 displays ER-α-mannosidase I activity and cleaves a single mannose residue from the B-branch of the oligomannosidic *N*-glycan (Figure 1A). The functionally redundant Golgi-α-mannosidases MNS1 and MNS2 act downstream of MNS3 and remove three mannose residues from the A- and C-branches (Figure 1B) (Liebminger et al., 2009). The resulting *N*-glycan (Man_5_GlcNAc_2_) is used by N-acetylglucosaminyltransferase I (GNTI) for the initiation of complex *N*-glycan processing in the *cis*/medial-Golgi (von Schaewen et al., 1993; Strasser et al., 1999).

In *Arabidopsis thaliana*, it is well documented that mutants deficient in the transfer of the lipid-linked oligosaccharide precursor or the first *N*-glycan processing reactions in the ER are lethal (Boisson et al., 2001; Gillmor et al., 2002; Koiwa et al., 2003; Lerouxel et al., 2005; Soussillane et al., 2009; Farid et al., 2011; Jeong et al., 2018). Moreover, *N*-glycan processing reactions in the Golgi apparatus generate complex *N*-glycans that are crucial for salt stress tolerance in *Arabidopsis* (Kang et al., 2008) and severely affect plant growth and reproduction in *Oryza sativa* (Fanata et al., 2013; Harmoko et al., 2016) and *Lotus japonicus* (Pedersen et al., 2017). Disruption of *MNS1* to *MNS3* genes in the *Arabidopsis mns123* triple mutant results in a severe vegetative growth defect with the formation of short and radially swollen roots (Liebminger et al., 2009). Glycoproteins from these plants carry almost exclusively Man_9_GlcNAc_2_ oligomannosidic *N*-glycans, but the molecular targets and underlying processes that are defective in the absence of mannose trimming are unknown (Strasser, 2014).

Recently, a study reported that mannose trimming reactions catalyzed by MNS1 and MNS2 play a crucial role for the salt stress tolerance of *Arabidopsis* (Liu et al., 2018). Under salt stress conditions, the stability of the heavily glycosylated endo-β1,4-glucanase KORRIGAN1 (RSW2) is compromised by pharmacologically inhibition of α-mannosidases or in the *mns12* double mutant. *Arabidopsis* RSW2 is involved in cellulose biosynthesis and has been linked to salt stress sensitivity in a previous study (Kang et al., 2008). Moreover, the genetic interaction between a temperature-sensitive *rsw2* allele and *N*-glycan processing mutants is well established (Kang et al., 2008; von Schaewen et al., 2008; Liebminger et al., 2009, 2010; Rips et al., 2014). In summary, these studies provide evidence for a link between *N*-glycan maturation, cellulose biosynthesis and the salt stress response of roots.

Despite their requirement for the full assembly of the lipid-linked oligosaccharide precursor, deficiency of ALG3(Henquet et al., 2008; Kajiura et al., 2010), ALG12 (Hong et al., 2009), or ALG9 (Hong et al., 2012) is tolerated by *Arabidopsis* and does not cause an obvious growth or developmental phenotype. Glycoproteins from these plants display only mild underglycosylation indicating that the mannose residues on the B- and C-branches are less critical for the overall *N*-glycosylation efficiency. Interestingly, an *Arabidopsis* mutant lacking a functional ALG9 partially suppresses the salt sensitivity of MNS1/MNS2-deficient plants (Liu et al., 2018). Whether the ALG3, ALG9, or ALG12 disruption can rescue the root growth defect of MNS-deficient plants under normal growth conditions has not been examined yet. Here, we investigated whether plants with defined defects in the biosynthesis of the B- and C-branches of the lipid-linked oligosaccharide precursor impact the vegetative growth phenotype of the *Arabidopsis mns123* mutant under non-stress conditions.

## Materials and Methods

### Plant Material

The *mns* mutants *(mns3* single, *mns12* double, and *mns123* triple mutant) were available from a previous study (Liebminger et al., 2009). The *os9* single mutant has been described previously (Hüttner et al., 2012). Homozygous *alg3* (SALK_064006) (Hüttner et al., 2014), *alg9* (GABI_831D07), and *alg12* (FLAG_310A12) (Hüttner et al., 2012) T-DNA insertion lines were identified by PCR from genomic DNA and crossed with *mns123* to generate the respective quadruple mutants. The *gntI* (SALK_073560, also called *cgl1-T*) T-DNA insertion mutant (Frank et al., 2008) was crossed with *alg3* and *mns3*, respectively, to obtain the *gntI alg3* and *gntI mns3* double mutants. The *os9* single mutant was crossed with *mns123* and *mns123 alg3* to obtain the quadruple mutant *mns123 os9* and the quintuple mutant *mns123 alg3 os9*. *Arabidopsis thaliana* wild-type and mutants were grown under long-day conditions (16-h-light/8-h-dark photoperiod) at 22°C. For mannosidase inhibitor treatments, seeds were directly germinated on 0.5 × Murashige and Skoog (MS) medium containing 2% sucrose and 20 μM kifunensine (Santa Cruz Biotechnology). For root length measurements, the different mutants were grown for 7 days on 0.5 × MS containing 1% sucrose. The seedlings were scanned and the primary root length was measured using Image J software. Four biological replicates were used to determine the root length. For quantification of rosette area and diameter (maximum distance between two points on the rosette boundary), pictures were taken from 23-day-old soil-grown plants and analyzed using Image J (Schneider et al., 2012).

### Immunoblotting and *N*-Glycan Analysis

Proteins were extracted from 7-day-old *Arabidopsis* seedlings with Laemmli sample buffer. The extracts were separated by SDS-PAGE and analyzed by immunoblotting with anti-horseradish peroxidase (HRP) antibodies to detect complex *N*-glycans carrying β1,2-xylose and core α1,3-fucose (Liebminger et al., 2009). The myrosinase TGG1 and the protein disulfide isomerase PDI5 were monitored using custom-made polyclonal antibodies as described in detail previously (Veit et al., 2015). GFP-tagged proteins were detected using an anti-GFP antibody (Roche). Endo H (New England Biolabs) digestions were done as described in detail recently from proteins of 12-day-old seedlings (Hüttner et al., 2012). *N*-linked glycan purification and matrix-assisted laser desorption ionization (MALDI) mass spectrometry (MS) were carried out as described (Liebminger et al., 2009).

### Cloning and Transformation of *mns123*

The constructs for complementation of the root growth phenotype of *Arabidopsis mns123* were generated in the following way. The cDNA region coding for the MNS1 (At1g51590) catalytic domain (CD: amino acids 89-560) was synthesized by GeneArt Gene Synthesis (Thermo Fisher Scientific) to remove an internal *Xba*I site and the *Bam*HI/*Bgl*II digested fragment was cloned into the *Bam*HI site of p47 (Hüttner et al., 2014) to generate p47-MNS1_CD_. The MNS1 promoter region was amplified by PCR from genomic DNA with 5′-TATAGGTACCGGTTGCTTTTCATCAATCTACCTAA-3′ and 5′-TATATCTAGATTCTCAACCCACTCAACAAAAAC-3′, *Kpn*I/*Xba*I digested and cloned into p47-MNS1_CD_ to generate p87-MNS1:MNS1_CD_. Next, the cDNA coding for the MNS1-CTS region (amino acids 1-88, CTS stands for the N-terminal cytoplasmic-transmembrane domain and stem region) (Liebminger et al., 2009) or the ST-CTS region (amino acids 1-52) (Boevink et al., 1998) were inserted into *Xba*I/*Bam*HI sites to generate p87-MNS1:MNS1_CTS_-MNS1_CD_ and p87-MNS1:ST_CTS_-MNS1_CD_, respectively. In the p87 vector, MNS1 is expressed from its endogenous promoter and carries a C-terminal GFP tag. Transgenic *Arabidopsis* were subsequently generated by floral dipping of *mns123* and selection on 0.5 × MS medium supplemented with hygromycin. The presence of the transgene was verified by PCR from genomic DNA using MNS1 and CTS-region specific primers.

To express MNS1 fused to mRFP in *N. benthamiana*, the full-length *Arabidopsis* MNS1 coding sequence was cloned into p48 (Hüttner et al., 2014) to generate p48-MNS1 following the same cloning strategy as outlined previously for p20-MNS1 (Schoberer et al., 2014). For expression of the ST-CTS-MNS1-catalytic domain fusion protein (ST-MNS1) under the control of the ubiquitin 10 promoter (p47 vector), the ST-CTS region was cloned into the *Xba*I/*Bam*HI site of p47-MNS1_CD_ resulting in the expression vector p47-ST-MNS1.

### Confocal Microscopy

Transient expression in *Nicotiana benthamiana* leaf epidermal cells was done by agrobacterium-mediated infiltration of leaves (Schoberer et al., 2009). For co-expression experiments, agrobacteria were diluted to an OD_600_ of 0.1 for p48-MNS1 (RFP-tagged full-length MNS1 expressed under the control of the ubiquitin 10 promoter) and pVKH18-En6:STmRFP (RFP-tagged ST-CTS region expressed under the control of the CaMV35S promoter) (Renna et al., 2005). An OD_600_ of 0.2 was used for p20-MNS1 (GFP-tagged full-length MNS1 expressed under the control of the CaMV35S promoter) and p47-ST-MNS1 (GFP-tagged fusion protein expressed under the control of the ubiquitin 10 promoter). Sampling and imaging of fluorescent proteins was performed 2 days after infiltration using a Leica TCS SP5 confocal microscope as described in detail previously (Schoberer et al., 2014). Postacquisition image processing was performed in Adobe Photoshop CS and Image J. For co-localization analysis, infiltrated leaf disks were (prior to image acquisition) treated for 30–45 min with the actin-depolymerizing agent latrunculin B (Merck Millipore) at a concentration of 25 μM to inhibit Golgi movement. Coefficients were calculated from selected Golgi stacks using Image J (Schneider et al., 2012) and the plugin JACoP (Bolte and Cordelières, 2006).

## Results

### *N*-Glycans Are Altered in *alg* Mutants With Defects in Mannose Trimming

To examine whether ALG-deficient mutants such as *alg3*, *alg9*, and *alg12* affect the *mns123* phenotype we generated *mns123 alg3*, *mns123 alg9*, and *mns123 alg12* quadruple mutants. Based on the known biosynthetic and processing reactions of these enzymes, it is predicted that all of the *Arabidopsis* mutants carry exclusively oligomannosidic *N*-glycans with a distinct structural composition (Supplementary Figure 1). In addition, we included the *gntI* T-DNA insertion mutant (Frank et al., 2008) and crosses of *gntI* with *mns3* (*gntI mns3* double mutant) as well as *gntI* crossed with *alg3* (*gntI alg3* double mutant) in our analysis because they produce distinct oligomannosidic *N*-glycans (von Schaewen et al., 1993; Henquet et al., 2008; Liebminger et al., 2009). To verify the absence of complex *N*-glycans in all the mutants, we extracted proteins from seedlings as well as rosette leaves and carried out immunoblotting with antibodies against β1,2-xylose and core α1,3-fucose residues that are characteristic for complex *N*-glycans (Strasser, 2014). As expected, none of the mutants displayed a signal showing the complete absence of complex *N*-glycans (Figure 1C and Supplementary Figure 2). Moreover, immunoblot analysis of the myrosinase TGG1 which is heavily glycosylated and carries mainly oligomannosidic *N*-glycans (Liebminger et al., 2012) or a protein disulfide isomerase (PDI) carrying two *N*-glycans revealed differences in mobility compared to the same proteins from wild-type (Figure 1D). We digested the protein extracts from the mutants displaying the fastest migrating TGG1 with endoglycosidase H (Endo H) to remove the oligomannosidic glycans and repeated the immunoblot. Upon digestion, the fastest migrating TGG1 was observed for *mns123 alg9*, *mns123*, and *gntI*. This band likely represents TGG1 with all oligomannosidic *N*-glycans removed (Figure 1E). Wild-type TGG1 appeared as a more diffuse band which can be explained by the presence of small amounts of Endo H-resistant complex-type *N*-glycans that lead to a slightly higher molecular weight and reduced mobility on immunoblots (Liebminger et al., 2012). Endo H digested TGG1 from *mns123 alg3* and *gntI alg3* displayed no clear shift in mobility compared to the undigested proteins indicating the presence of altered oligomannosidic *N*-glycans (Figure 1E).

**FIGURE 2:**
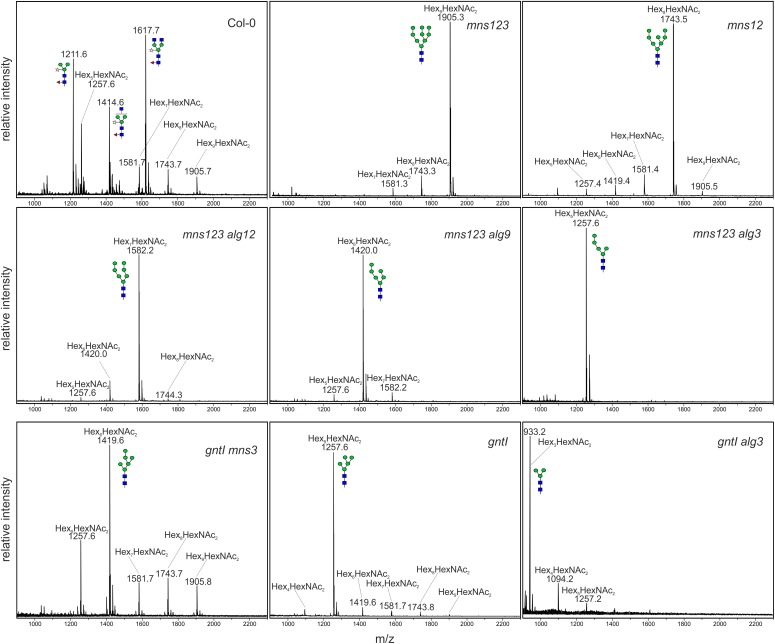
MALDI MS analysis of *N*-glycans isolated from rosette leaves of 5-week-old wild-type (Col-0) or different *Arabidopsis* mutants.

To determine the *N*-glycan structures in the mutants more precisely, we harvested 500 mg leaves from 5-week-old soil-grown plants, purified the *N*-glycans and analyzed them by MALDI MS. In accordance with immunoblot data, the major peak corresponds to a single mannosidic *N*-glycan in all mutants (Figure 2). Whereas in wild-type Col-0, in *alg3* (Henquet et al., 2008), *alg12*, and *alg9* (Supplementary Figure 3) single mutants the majority of *N*-glycans are of the complex or paucimannosidic-type, the different mutants displayed Man_9_GlcNAc_2_ (*mns123*), Man_8_GlcNAc_2_ (*mns12*), Man_7_GlcNAc_2_ (*mns123 alg12*), Man_6_GlcNAc_2_ (*mns123 alg9*), Man_5_GlcNAc_2_ (*mns123 alg3*), Man_6_GlcNAc_2_ (*gntI mns3*), Man_5_GlcNAc_2_ (*gntI*), and Man_3_GlcNAc_2_ (*gntI alg3*) structures. Thus, the MALDI MS data are consistent with the immunoblot data and confirm that the biosynthesis and processing defects cause the proposed alteration of *N*-glycan structures as outlined in Supplementary Figure 1.

### Deficiency of ALGs Rescues the Root Growth Defect of *mns123*

**FIGURE 3:**
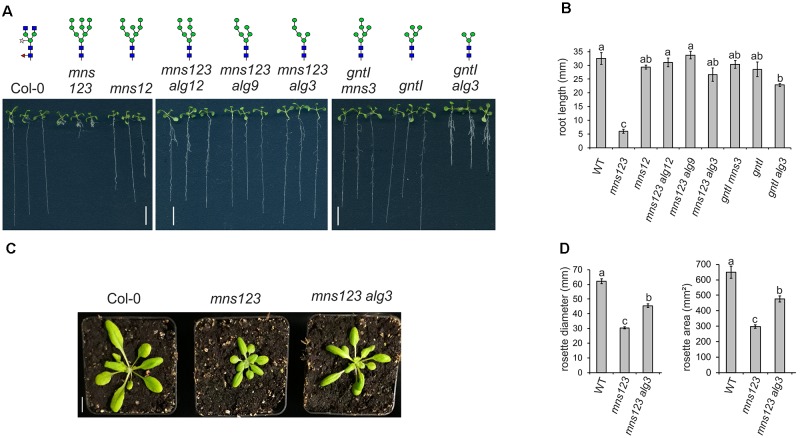
**(A)** The root growth phenotype of 9-day-old *Arabidopsis* seedlings with defects in lipid-linked oligosaccharide precursor biosynthesis and/or *N*-glycan processing. Seedlings were grown vertically on 0.5 × MS containing 2% sucrose. The major *N*-glycan structures in these lines are indicated as illustrations. Scale bar = 1 cm. **(B)** The primary root length of WT and the different mutants when grown for seven days on 0.5 × MS containing 1% sucrose are shown. Data represent mean values ± standard error. Data were analyzed using one-way ANOVA with Tukey’s *post hoc-*test (four biological replicates, 25–110 seedlings/each). Different letters indicate significant differences among different genotypes (*P* < 0.05). **(C)** Phenotype of 4-week-old soil grown *Arabidopsis* Col-0, *mns123*, and *mns123 alg3.* Scale bar = 1 cm. (**D**) Quantification of the maximum rosette diameter and the rosette area of 23-day-old plants. Data represent mean values ± standard error (*n* ≥ 27 plants). Data were analyzed using one-way ANOVA with Tukey’s *post hoc*-test. Different letters indicate significant differences among different genotypes (*P* < 0.01).

To investigate the impact of altered *N*-glycan structures and impaired mannose trimming on plant growth, we compared the primary root length of all characterized mutants (Figures 3A,B and Supplementary Figure 4). Intriguingly, the previously described severe root growth defect (Liebminger et al., 2009) was only found for *mns123* containing unprocessed Man_9_GlcNAc_2_ structures. The *mns123 alg12*, *mns123 alg9*, and *mns123 alg3* quadruple mutants displayed a wild-type like root phenotype indicating that the absence of mannose residues at the B- or C-branches of the *N*-glycans rescues the *mns123* root growth phenotype. The primary root of the *gntI alg3* double mutant was also significantly shorter compared to wild-type but clearly less affected than *mns123*. A suppression of the *mns123* phenotype was also seen when *mns123 alg3* plants were grown on soil (Figure 3C) or on MS-medium in the dark (Supplementary Figure 4). The rosette area and the maximum rosette diameter of *mns123 alg3* were smaller than wild-type, but significantly larger than *mns123* (Figure 3D). A suppression of the *mns123* phenotype was detected for *mns123 alg12* and *mns123 alg9* (data not shown).

**FIGURE 4:**
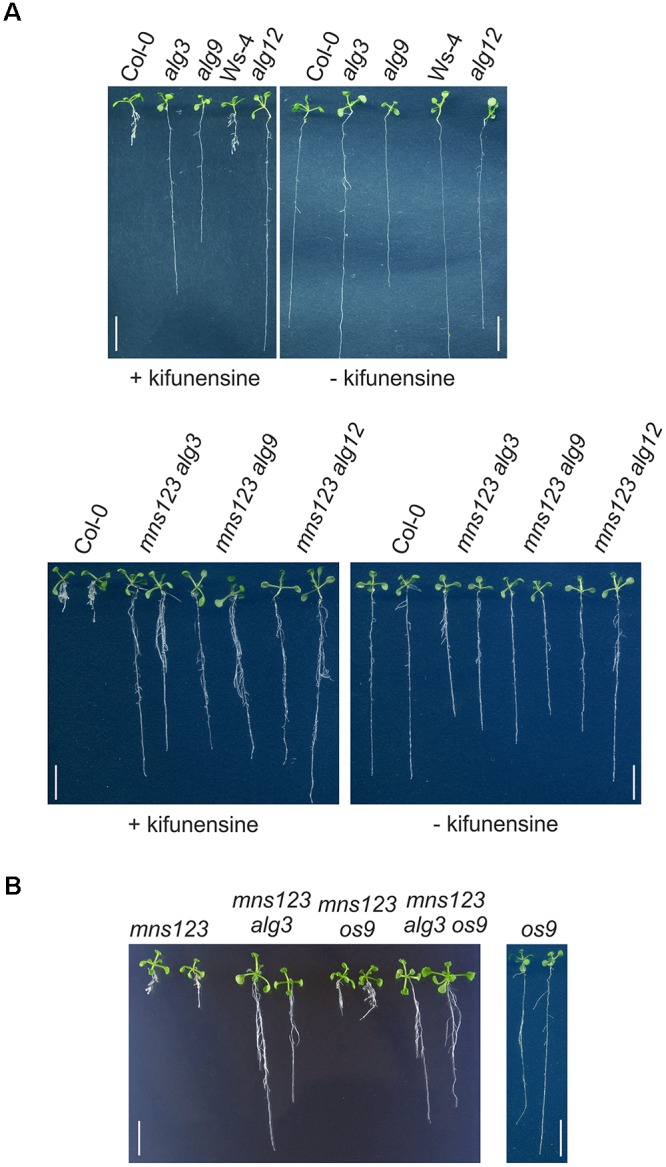
**(A)** Root phenotype of *Arabidopsis* Col-0, Ws-4 (parental line for *alg12*), different *alg* single mutants, and the *mns123 alg* quadruple mutants in the presence of kifunensine. Seedlings were grown for 10 days on 0.5 × MS containing 2% sucrose in the presence or absence of 20 μM kifunensine. Scale bar = 1 cm. **(B)** Root phenotype of 14-day-old *Arabidopsis* seedlings grown on 0.5 × MS supplemented with 1% sucrose. Scale bar = 1 cm.

Next, we assessed the response of *alg3*, *alg9*, and *alg12* seedlings to kifunensine, a specific class I α-mannosidase inhibitor (Elbein et al., 1990). In a previous study, we have shown that kifunensine blocks MNS1 to MNS3-mediated mannose trimming and leads to a root growth defect of wild-type seedlings that is reminiscent of the *mns123* phenotype (Liebminger et al., 2009). In contrast to wild-type, the root growth of the three *alg* single mutants appeared insensitive to pharmacological inhibition of α-mannosidases (Figure 4A). Likewise, the quadruple mutants displayed long roots in the presence of kifunensine. Taken together, these data are consistent with the observed suppression of the *mns123* phenotype in the ALG-deficient mutants.

### Suppression of the Root Growth Defect Is Uncoupled From Glycan-Dependent ERAD

Kifunensine is also used as specific inhibitor for ER-associated degradation (ERAD) of misfolded glycoproteins (Hüttner et al., 2012) and *alg9* as well as *alg12* have been identified as specific suppressors of ERAD in *Arabidopsis* (Hong et al., 2009, 2012). Therefore, we examined whether the observed rescue of the *mns123* growth phenotype is caused by an inhibition of the ERAD pathway. To this end, we crossed *mns123* with the *os9* mutant lacking a functional variant of the carbohydrate binding protein OS9 (Hüttner et al., 2012). Together with other proteins, OS9 is part of the HRD1 complex that selects aberrant glycoproteins to glycan-dependent ERAD. The *mns123 os9* quadruple mutant displayed the same root growth defect like *mns123* and the *mns123 alg3 os9* quintuple mutant phenocopied *mns123 alg3* showing that the observed phenotypic suppression is independent of a functional ERAD pathway (Figure 4B).

### Golgi-Mediated Mannose Trimming Rescues the Root Growth Defect

**FIGURE 5:**
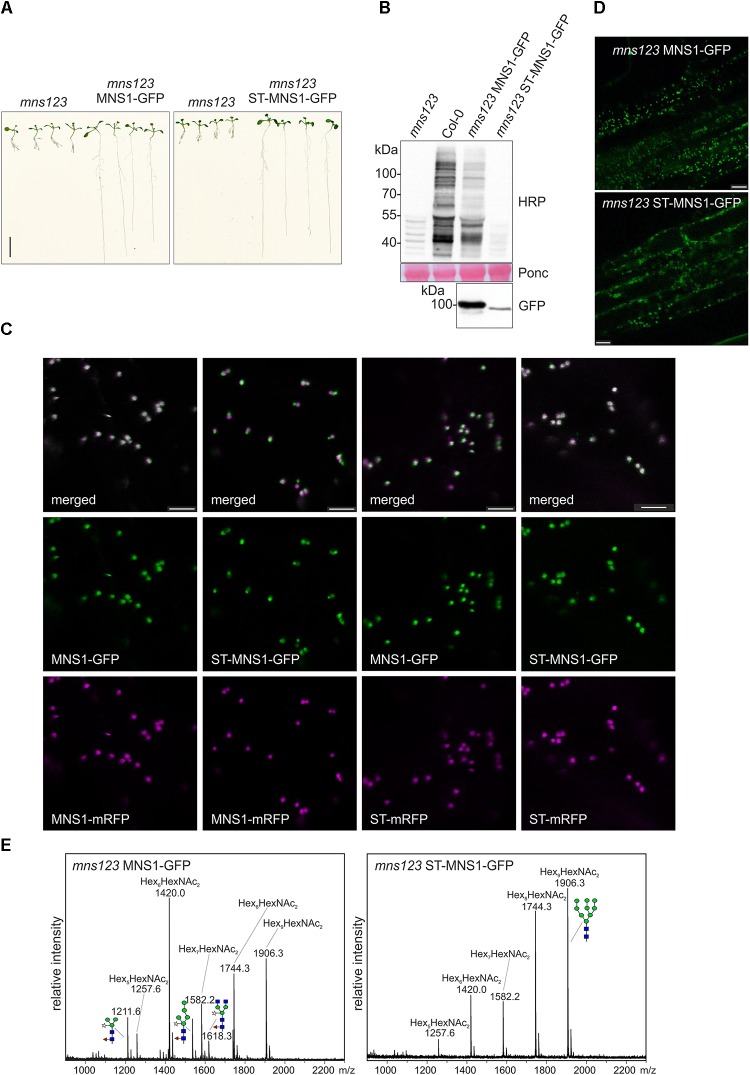
**(A)** Seedlings grown on 0.5 × MS medium containing 1% sucrose for 9 days. Scale bar = 1 cm. **(B)** Immunoblot analysis using anti-horseradish peroxidase (HRP) antibodies, which recognize β1,2-xylose and core α1,3-fucose residues on *N*-glycans. Ponceau S (Ponc) staining of the membrane is shown as a loading control and anti-GFP antibody was used to monitor MNS1-GFP and ST-MNS1-GFP expression. **(C)** Confocal images (2 days after infiltration) of *N. benthamiana* leaf epidermal cells transiently co-expressing MNS1-mRFP or ST-mRFP with MNS1-GFP or ST-MNS1-GFP. The white color in the merged image shows the co-localization. Scale bare = 5 μm. **(D)** Confocal images from roots of 10-day-old *Arabidopsis mns123* seedlings expressing MNS1-GFP or ST-MNS1-GFP. Scale bar = 10 μm. **(E)** Leaves from different transgenic lines were pooled and subjected to MALDI MS analysis. The characteristic oligomannosidic *N*-glycan (Hex_5-9_HexNAc_2_) peaks are indicated. Man_9_GlcNAc_2_ and different complex/truncated *N*-glycans with xylose and fucose are shown as illustrations.

To investigate whether the observed *mns123* root growth phenotype is caused by the inability to process *N*-glycans in the Golgi apparatus, we expressed MNS1-GFP under its own promoter with its endogenous *cis*/medial-Golgi targeting and retention signal (MNS1-CTS region) (Liebminger et al., 2009) in the *mns123* mutant. MNS1-GFP expression restored the formation of complex *N*-glycans and normal root growth showing that this transgene is functional (Figures 5A,B and Supplementary Figure 5).

Next, we examined whether MNS1 targeting to the *trans*-Golgi can rescue the root growth phenotype of *mns123*. To this end, we replaced the N-terminal MNS1-CTS region with the well-known *trans*-Golgi targeting signal from rat α2,6-sialyltransferase (ST-CTS region) (Boevink et al., 1998). We assumed that the ST-MNS1 fusion protein is targeted to the *trans*-Golgi because the CTS-region is typically the determinant for sub-Golgi compartmentation of *N*-glycan processing enzymes in plants (Saint-Jore-Dupas et al., 2006; Schoberer et al., 2013, 2014). To monitor the subcellular localisation we transiently co-expressed ST-MNS1-GFP with marker proteins in *N. benthamiana* leaf epidermal cells. ST-MNS1-GFP was co-expressed with the *cis*/medial-Golgi protein MNS1-mRFP (Liebminger et al., 2009; Nebenführ et al., 1999) and the *trans*-Golgi marker ST-mRFP (Boevink et al., 1998), respectively. Confocal microscopy images of ST-MNS1-GFP and ST-mRFP displayed overlapping signals (Figure 5C) indicating that these proteins reside in the same Golgi cisternae. By contrast, Golgi stacks clearly appeared tricolored when ST-MNS1-GFP was co-expressed with MNS1-GFP indicating distinct intra-Golgi distributions and *trans*-Golgi accumulation of ST-MNS1-GFP. Co-localization analyses of ST-MNS1-GFP with ST-mRFP corroborated these findings (Table 1). In *Arabidopsis* seedlings, MNS1-GFP and ST-MNS1-GFP labeled mobile structures resembling Golgi stacks (Figure 5D). Compared to MNS1-GFP, the fluorescence signal was much lower for ST-MNS1-GFP expressing plants and ST-MNS1-GFP protein levels were reduced (Figure 5B). The lower amounts of ST-MNS1-GFP may be explained by the secretion of excess ST-fusion protein from the Golgi (Runions et al., 2006). Nonetheless, ST-MNS1-GFP expression resulted in suppression of the *mns123* root growth defect (Figure 5A). Immunoblot and MALDI MS analyses revealed the absence of complex *N*-glycans in ST-MNS1-GFP expressing *mns123* (Figures 5B,E). However, a considerable amount of Man_9_GlcNAc_2_ *N*-glycans was processed to Man_8_GlcNAc_2_ and other oligomannosidic structures. In summary, these data indicate that removal of mannose residues in a late Golgi compartment is sufficient to rescue the root growth defect.

**Table 1:** Co-localization of MNS1-GFP and ST-MNS1-GFP with the *trans*-Golgi marker ST-mRFP in *N. benthamiana* leaf epidermal cells.

Combinations		Manders’ co-localization coefficient
MNS1-GFP	ST-mRFP	0.56 ± 0.06
ST-MNS1-GFP	ST-mRFP	0.87 ± 0.05

		**Pearson’s correlation coefficient**

MNS1-GFP	ST-mRFP	0.82 ± 0.03
ST-MNS1-GFP	ST-mRFP	0.94 ± 0.02


*Mean values ± standard deviation are shown. *P* value < 0.001 (Student’s t-test). Measurements were made on 50 Golgi stacks from 10 separate cells with Image J and the plugin JACoP.*

## Discussion

Impaired mannose trimming from oligomannosidic *N*-glycans results in a severe root phenotype and subsequent growth defects of aerial rosettes (Liebminger et al., 2009). The underlying process(es) and affected glycoproteins have not been discovered yet. Plants contain an entire set of ALG glycosyltransferases to synthesize the lipid-linked oligosaccharide precursor composed of 14 sugars. The previously described *alg10* knockout plants display a leaf growth defect (Farid et al., 2011) and *lew3* (defect in the *Arabidopsis ALG11* gene) null mutants are embryo lethal (Zhang et al., 2009). By contrast, ALG3, ALG9, and ALG12-deficient *Arabidopsis* display a wild-type like growth despite the fact that they have altered oligomannosidic *N*-glycans (Henquet et al., 2008; Hong et al., 2009, 2012). Even under salt/osmotic stress conditions, the root growth of *alg3* is comparable to wild-type (Kajiura et al., 2010) suggesting that the absence of mannose residues on the B- and C-branches of the assembled oligosaccharide is well tolerated by the plants and does not severely interfere with developmental processes. In agreement with these findings, our data show that ALG3, ALG9, and ALG12 deficiency rescue the growth phenotype of *mns123*. The removal of mannose residues from the B- and C-branches is not only crucial for salt stress tolerance as shown recently (Liu et al., 2018), but also for normal growth. Liu and colleagues reported that RSW2 stability is affected under salt stress conditions when mannose trimming is blocked on the C-branch (Liu et al., 2018). The authors proposed that the unprocessed α1,2-mannose residue on the C-branch is recognized by an as yet unknown carbohydrate binding protein which may divert glycoproteins like RSW2 for degradation under salt stress conditions. We did not examine the fate of RSW2 under our growth conditions because previous studies have shown that *N*-glycan processing does not have a direct effect on RSW2 function (Liebminger et al., 2013; Rips et al., 2014). Moreover, the *mns12* double mutant with the unprocessed C-branch displays a much less severe root phenotype compared to *mns123*. Based on these data, it is likely that the salt stress sensitivity and the root phenotype observed under normal growth conditions involve different glycoproteins and processes. Whether specific lectin-like receptors recognize glycoproteins with unprocessed terminal mannose residues at the B- and/or C-branches remains to be shown in future studies. Our data suggest that such lectins would bind with the highest affinity to Man_9_GlcNAc_2_ while *N*-glycans lacking a terminal α1,2-mannose residue on either the B (e.g., *mns12*) or C-branch (e.g., *mns123 alg12*) display reduced interaction.

In the ER, a glycan-dependent ERAD pathway removes misfolded glycoproteins. This process involves the generation of a conserved glycan signal with an exposed terminal α1,6-mannose residue on the C-branch that is subsequently recognized by the lectin OS9. The binding of OS9 selects substrates for specific degradation via the SEL1L-HRD1 complex (Clerc et al., 2009; Su et al., 2011; Hong et al., 2012; Hüttner et al., 2014). OS9 is a mannose 6-phosphate receptor homology (MRH) domain-containing protein that resides in the ER in plants. Here, we provide genetic evidence that OS9 and glycan-dependent ERAD can be uncoupled from the root growth defect. We generated a chimeric ST-MNS1 variant that was targeted to the *trans*-Golgi in *N. benthamiana* leaf epidermal cells and rescued the *mns123* root growth defect. Although not directly confirmed by co-localization in seedlings, we propose that the ST-MNS1 variant is located in the *trans*-Golgi in *Arabidopsis* as shown previously for full-length ST (Wee et al., 1998). The late Golgi-targeting in *Arabidopsis* is supported by the absence of complex *N*-glycans in ST-MNS1-GFP expressing plants. Importantly, ST-MNS1-GFP expression resulted in the cleavage of mannose residues from oligomannosidic *N*-glycans and the suppression of the *mns123* root phenotype indicating that a late Golgi or post-Golgi event is abolished in the absence of mannose trimming. Such an event could be a specific recognition by a mannose-binding lectin that directs glycoproteins with unprocessed oligomannosidic *N*-glycans to a degradation pathway. Alternatively, a biologically relevant protein–protein interaction could be affected in the *mns123* mutant due to the presence of Man_9_GlcNAc_2_ *N*-glycans leading to the observed growth phenotype.

In mammals, MRH domain-containing lectins are found in other parts of the secretory pathway including the Golgi. Binding of MRH-domain containing receptors to mannose-6-phosphate from *N*-glycans of cargo glycoproteins leads to their transport from the *trans*-Golgi network (TGN) to the lysosome (Castonguay et al., 2011). MRH domain-containing receptors implicated in targeting of lysosomal/vacuolar enzymes have been described in *Drosophila* and yeast. Plants, however, lack homologs of these receptors (de Marcos Lousa and Denecke, 2016). Besides OS9, the only known MRH domain-containing protein in plants is the β-subunit from α-glucosidase II that resides in the ER and modulates the *N*-glycan processing activity of the α-glucosidase II α-subunit (Lu et al., 2009; Olson et al., 2013). Other lectin-types such as mammalian ERGIC-53 and VIP36 are known to bind to oligomannosidic glycans of cargo glycoproteins and are involved in their transport from the ER to the ER-Golgi intermediate compartment (ERGIC) or *cis*-Golgi (Hauri et al., 2000). The *Arabidopsis* genome does not contain homologs of these proteins and similar cargo receptors involved in ER to Golgi transport have not been described in plants. On the other hand, *Arabidopsis* has numerous uncharacterized lectin domain containing proteins like lectin receptor kinases that could be involved in binding to specific oligomannosidic *N*-glycans (Eggermont et al., 2017; Teixeira et al., 2018). For example, *Galanthus nivalis* agglutinin (GNA) binds preferentially to terminal α1,3-linked mannose residues and to a lesser extend also to α1,6-linked mannose containing structures (Shibuya et al., 1988). *Arabidopsis* contains 49 putative lectin genes coding for proteins with a GNA domain and almost all of them have either a signal peptide sequence or transmembrane domain (Eggermont et al., 2017) which makes them potential candidates for lectins binding to oligomannosidic *N*-glycans in the secretory pathway.

In addition to regulation of lectin binding events, the presence or absence of certain mannose residues on *N*-glycans of secretory proteins may directly affect the protein conformation and consequently the interaction with other proteins. Plant soluble vacuolar proteins are typically sorted by specific vacuolar sorting receptors (VSRs) that recognize amino acid motifs from cargo proteins. *Arabidopsis* VSR1 is glycosylated with complex *N*-glycans and *N*-glycosylation stabilizes its ligand binding conformation (Shen et al., 2014). Moreover, there is evidence that VSRs can bind their cargo already in the ER where the VSR *N*-glycans would still be oligomannosidic (Künzl et al., 2016). It is therefore tempting to speculate that the cargo-binding affinity of glycosylated VSRs such as VSR1 is not only influenced by *N*-glycosylation but also by a distinct *N*-glycan structure. In this scenario, unprocessed oligomannosidic *N*-glycans could provide stronger cargo binding than partially processed oligomannosidic or complex *N*-glycans. The stepwise ER to *trans*-Golgi mediated processing of *N*-glycans could represent an elegant mechanism to alter cargo affinity and enable the controlled cargo release on their route to the vacuole. Whether the VSR *N*-glycan composition has indeed an impact on cargo binding and how such a glycan-mediated process can be integrated with our findings and with recent data showing that recycling VSRs bind cargo in the *cis*-Golgi (Früholz et al., 2018) remains to be shown in future studies.

## Author Contributions

CV, JK, FA, and RS have made a substantial and intellectual contribution to the work and approved it for publication.

## Conflict of Interest Statement

The authors declare that the research was conducted in the absence of any commercial or financial relationships that could be construed as a potential conflict of interest.
