# Supplementary material for: Processing of the Terminal Alpha-1,2-Linked Mannose Residues From Oligomannosidic N-Glycans Is Critical for Proper Root Growth
Source: Front Plant Sci. 2018 Dec 6;9:1807. doi: 10.3389/fpls.2018.01807 (PMC6291467; doi:10.3389/fpls.2018.01807)
Supplement: Supplementary file 1 [file Data_Sheet_1.PDF]

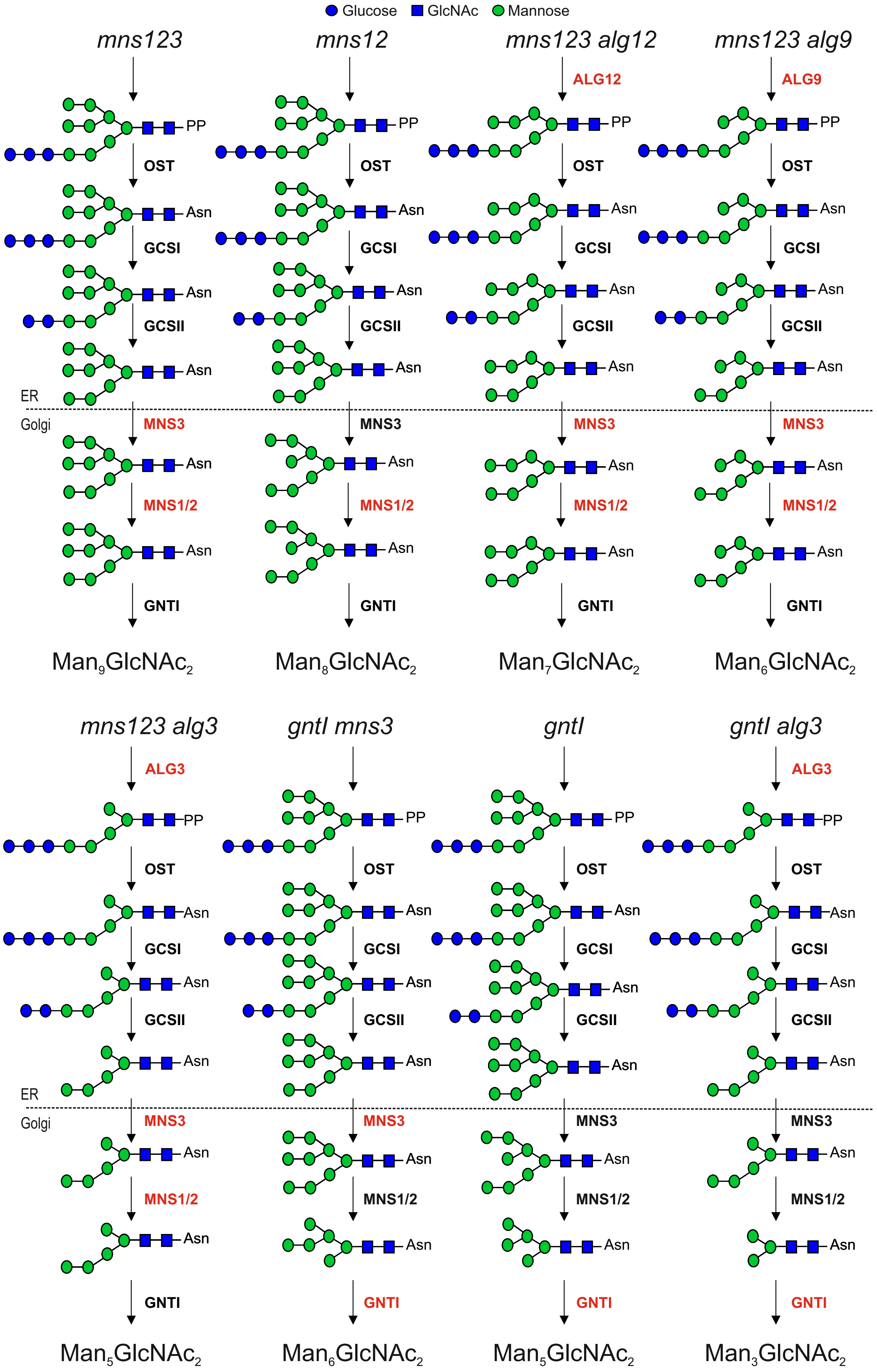

Supplemental Figure 1. Illustration of *N*-glycan processing pathways in the analysed *Arabidopsis* mutants. The blocked biosynthesis (*alg* mutants) or processing defects are highlighted in red. For abbreviations of enzymes see Figure 1.

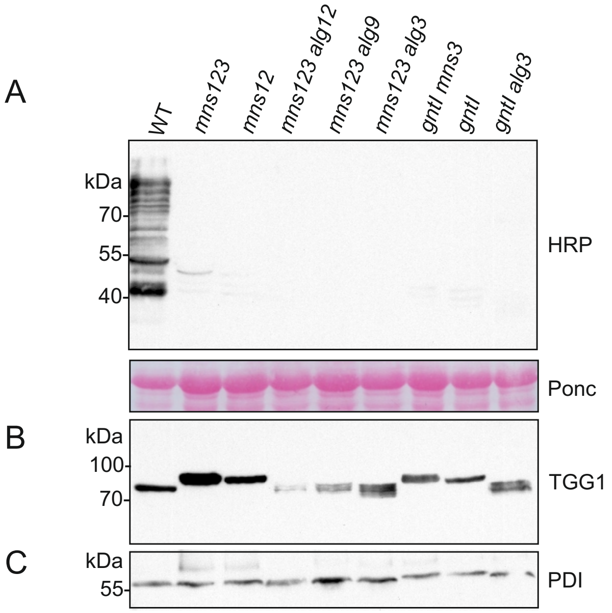

Supplemental Figure 2. Immunoblot analysis. Proteins were extracted from rosette leaves of 4-week-old *Arabidopsis* with Laemmli sample buffer. (A) The extracts were separated by SDS-PAGE and analysed by immunoblotting with anti-horseradish peroxidase (HRP) antibodies to detect complex *N*-glycans. A positive signal is only observed in wild-type Col-0 (WT) carrying processed complex *N*-glycans with β1,2-xylose and core α1,3-fucose residues. Ponceau S (Ponc) staining of the membrane is shown as a loading control. (B) The same extracts were analysed with antibodies against the myrosinase TGG1 or (C) protein disulfide isomerase (PDI).

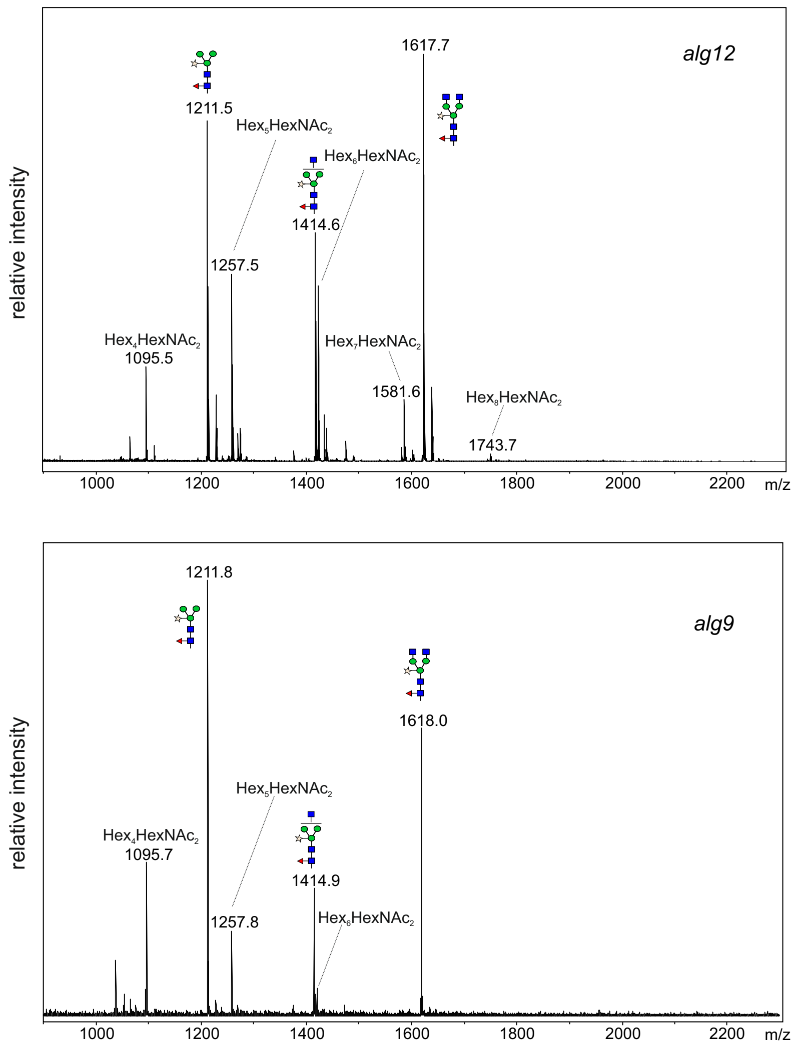

Supplemental Figure 3. MALDI MS analysis of *N*-glycans isolated from rosette leaves of 5-week-old *alg12* and *alg9 Arabidopsis* mutants.

A

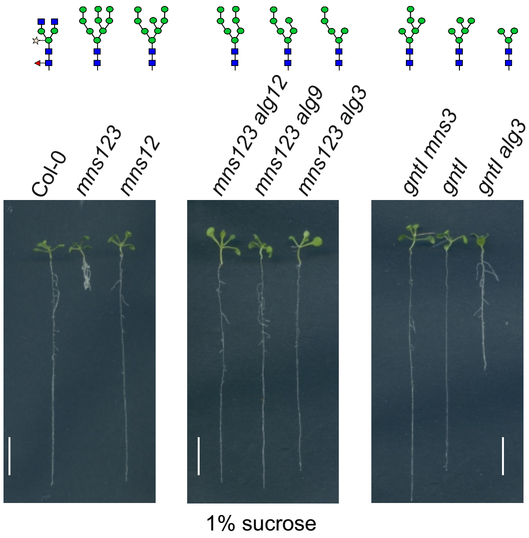

B

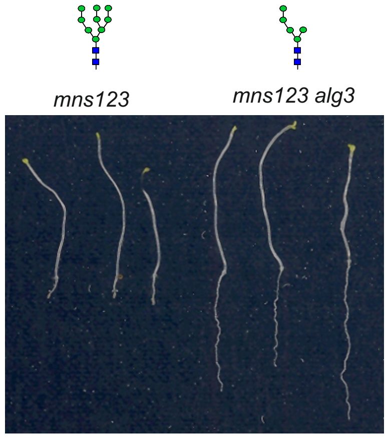

Supplemental Figure 4. (A) Growth of *Arabidopsis* seedlings with defects in lipid-linked oligosaccharide precursor biosynthesis and/or *N*-glycan processing on 0.5 x MS + 1% sucrose for 9 days. The major *N*-glycan structures in these lines are indicated as illustrations. Scale bar = 1 cm. (B) Growth phenotype of *mns123* and *mns123 alg3* grown for 12 days on 0.5 x MS + 1% sucrose in the dark.

Figure 1C

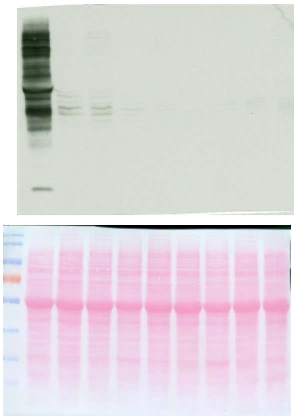

Figure 1D top

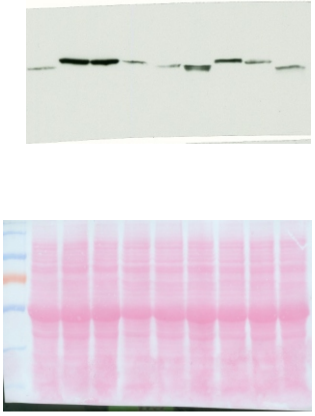

Figure 1D bottom

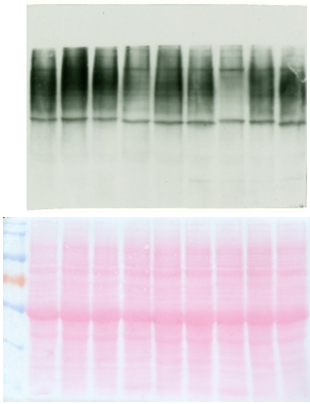

Figure 1E

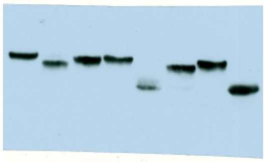

Figure 5B top

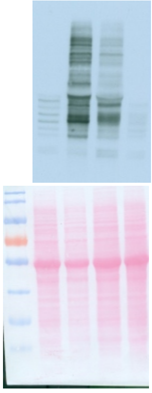

Figure 5B bottom

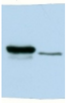

Suppl. Fig. 2A

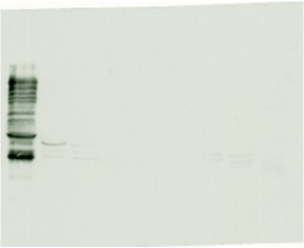

Suppl. Fig. 2A

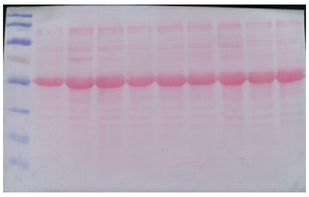

Suppl. Fig. 2B

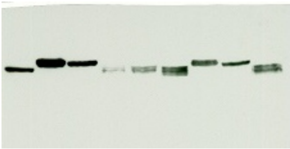

Suppl. Fig. 2C

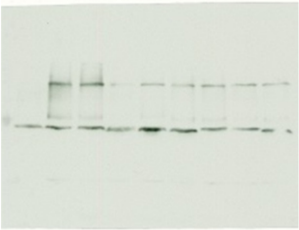

Supplemental Figure 5. Uncropped images of the blots shown in Figure 1, Figure 5 and Supplemental Figure 2.
